# Liposomal Piceatannol Mitigates Methotrexate-Induced Oxidative Renal Injury via Modulation of Nrf2/HO-1, TLR4/NF-κB, MAPK, and Apoptotic Pathways in Rats

**DOI:** 10.3390/biom16040517

**Published:** 2026-03-31

**Authors:** Fahad Alshammari, Ekramy M. Elmorsy, Abdulrahman S. Aldaghmi, Fahd Alaajam, Ahmed S. Shams, Gehad E. Elshopakey, Manal S. Fawzy, Nora Hosny

**Affiliations:** 1 Department of Biology, College of Science, Jouf University, Sakaka 72341, Saudi Arabia; fahadm@ju.edu.sa (F.A.); asaldaghmi@ju.edu.sa (A.S.A.); 2 Center for Health Research, Northern Border University, Arar 73213, Saudi Arabia; ekramy.elmorsy@nbu.edu.sa; 3 Department of Medical Laboratory Technology, College of Nursing and Health Science, Jazan University, Jazan 45142, Saudi Arabia; falaajam@jazanu.edu.sa; 4 Department of Human Anatomy and Embryology, Faculty of Medicine, Suez Canal University, Ismailia 41522, Egypt; ashams@umn.edu; 5 Department of Clinical Pathology, Faculty of Veterinary Medicine, Mansoura University, Mansoura 35516, Egypt; gehadelshopakey@mans.edu.eg; 6 Medical Biochemistry and Molecular Biology Department, Faculty of Medicine, Suez Canal University, Ismailia 41522, Egypt; nora_hosny@med.suez.edu.eg

**Keywords:** methotrexate, piceatannol, liposomal nanoparticles, oxidative stress, nephroprotection, Nrf2/HO-1 pathway, TLR4/NF-κB, MAPK signaling

## Abstract

Methotrexate (MTX) is a widely used chemotherapeutic and immunosuppressive agent, but its clinical utility is limited by oxidative stress-mediated renal toxicity. This study evaluated the nephroprotective potential of the dietary polyphenolic bioactive molecule piceatannol (PIC) in its crude and liposomal nanoparticle (PIC-LNP) forms against MTX-induced kidney injury in rats. Sixty rats were allocated into six groups and received vehicle, PIC, PIC-LNPs, MTX, or combinations of MTX with PIC or PIC-LNPs. MTX administration induced marked renal dysfunction and oxidative/nitrosative stress, reflected by elevated serum urea, creatinine, and uric acid, together with increased renal ROS, MDA, protein carbonyls, 8-OHdG, and nitric oxide, in parallel with suppression of the Nrf2/HO-1 antioxidant pathway. These disturbances were accompanied by activation of TLR4/NF-κB and MAPK signaling, upregulation of pro-inflammatory cytokines, and a shift toward apoptosis, as evidenced by increased Bax and caspase-3 and reduced Bcl-2 expression. Histological and ultrastructural analyses confirmed extensive glomerular and tubular damage with mitochondrial disruption and cytoplasmic vacuolations. PIC treatment attenuated these MTX-induced alterations, whereas the liposomal formulation conferred superior protection. PIC-LNPs restored Nrf2/HO-1 signaling, enhanced endogenous antioxidant defenses, reduced oxidative/nitrosative and inflammatory responses, and normalized apoptotic markers, accompanied by substantial preservation of renal architecture and cellular integrity. Immunohistochemistry demonstrated strong Nrf2 expression with minimal NF-κB activation in the PIC-LNP group. Collectively, these findings highlight liposomal piceatannol as a promising bioactive-molecule-based strategy for controlling oxidative stress and mitigating chemotherapy-associated oxidative-stress-related renal injury.

## 1. Introduction

Methotrexate (MTX) is widely used in clinical practice owing to its immunosuppressive, anti-inflammatory, and anti-proliferative properties, and it remains integral to many autoimmune and anticancer regimens [1,2]. However, its therapeutic utility is constrained by dose-dependent nephrotoxicity, which becomes a major limitation during long-term or intensive therapy [3,4]. MTX-induced renal injury is attributed to intratubular drug deposition, obstruction of urine flow, and reduced glomerular filtration, culminating in elevated serum urea, creatinine, and uric acid, and increasing the risk of both acute and chronic kidney injury [5,6].

The mechanisms underlying MTX nephrotoxicity are multifactorial, but oxidative stress is considered a central driver. MTX promotes excessive reactive oxygen species (ROS) generation, leading to lipid peroxidation, protein oxidation, and genotoxic DNA damage, together with depletion of enzymatic antioxidants such as superoxide dismutase (SOD) and catalase (CAT), and non-enzymatic defenses including reduced glutathione [4,7,8]. MTX-induced ROS accumulation also impairs mitochondrial function, favors cytochrome c release, disrupts the Bax/Bcl-2 balance, and activates caspase-3 in tubular epithelial cells, thereby promoting apoptosis and progressive renal injury [9]. In parallel, MTX activates redox-sensitive transcription factors, such as NF-κB, leading to increased production of pro-inflammatory cytokines, leukocyte infiltration, and edema, which further exacerbate tubular damage and fibrotic remodeling. Stress-activated kinases, including p38 MAPK, JNK, and ERK, further amplify inflammatory and apoptotic responses, underscoring the tight interplay between oxidative stress, inflammation, and cell death in MTX-induced kidney injury [10,11].

These insights have stimulated interest in natural polyphenols as multitarget modulators of oxidative and inflammatory pathways in nephrotoxicity. Piceatannol (PIC), a hydroxylated stilbene derivative, has emerged as a promising candidate because of its potent antioxidant, anti-inflammatory, and anti-apoptotic activities [12,13]. PIC has been reported to attenuate ROS generation, preserve mitochondrial membrane potential, suppress pro-inflammatory mediators, and regulate the Bax/Bcl-2/caspase-dependent apoptotic pathway [14]. Collectively, these properties suggest that PIC could counteract the oxidative, inflammatory, and apoptotic components of MTX-induced renal damage.

Despite these benefits, the clinical translation of PIC is hindered by chemical instability, poor aqueous solubility, rapid metabolism, and low oral bioavailability, all of which limit its effective accumulation in target tissues [15]. Liposomal nanoparticles offer a rational strategy to overcome these drawbacks. Liposomes are spherical phospholipid vesicles capable of encapsulating both hydrophilic and hydrophobic compounds, thereby improving their stability and solubility [16]. Encapsulation of PIC in nanocarriers can protect it from degradation, prolong its circulation time, enhance tissue targeting, and allow for controlled, sustained release, ultimately increasing its bioavailability and therapeutic efficacy [17,18,19].

In this context, the present study examined whether PIC encapsulated in liposomal nanoparticles (PIC-LNPs) affords superior protection against MTX-induced renal injury compared with crude PIC. We specifically focused on the capacity of PIC and PIC-LNPs to modulate oxidative stress, inflammatory responses, and apoptotic signaling in an oxidative stress-driven nephrotoxicity model, thereby exploring a bioactive molecule-based nanotherapeutic strategy to mitigate chemotherapy-associated kidney injury.

## 2. Materials and Methods

### 2.1. Drugs

Methotrexate (MTX; 50 mg/5 mL injectable solution) was obtained from EIPICO Pharmaceutical Company, Tenth of Ramadan City, Egypt. Piceatannol (PIC, ≥98%), cholesterol, and L-α-phosphatidylcholine (L-PC) were purchased from Sigma-Aldrich, Cairo, Egypt, and dissolved in DMSO (Sigma-Aldrich, Cairo, Egypt) immediately before use to ensure complete solubilization. Piceatannol-loaded liposomal nanoparticles (PIC-LNPs) were prepared as described below. The MTX dose was selected according to [20], while the PIC dose was within the previously reported safe and effective range [21].

### 2.2. Preparation of Piceatannol-Loaded Liposomal Nanoparticles (PIC-LNPs)

**Figure 1:**
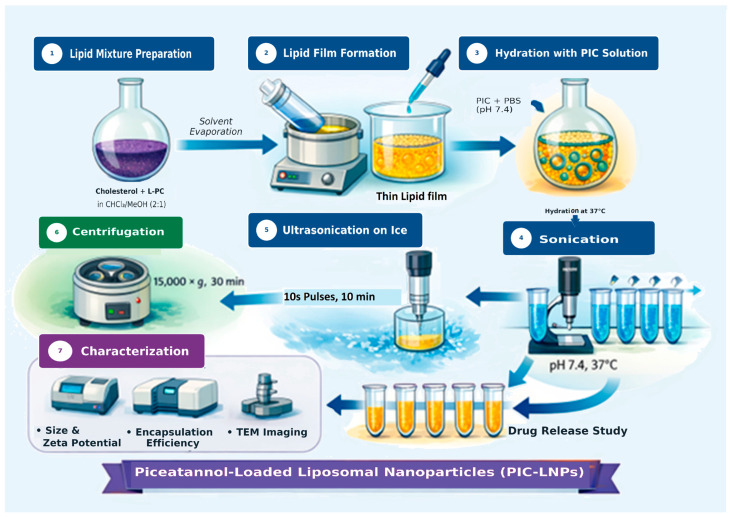
Schematic representation of PIC-LNP preparation by thin-film hydration, sonication, and purification, followed by physicochemical characterization (particle size, zeta potential, TEM).

PIC-LNPs were prepared by the thin-film hydration method (Figure 1). L-PC (70 mg; 0.09 mmol) and cholesterol (30 mg; 0.078 mmol) were dissolved in 10 mL chloroform: methanol (2:1, *v*/*v*) in a round-bottom flask to obtain a clear lipid solution (10 mg/mL). Organic solvents were removed under reduced pressure to form a thin, uniform lipid film. The film was hydrated with 5 mL phosphate-buffered saline (PBS; pH 7.4; VWR Chemicals, Radnor, PA, USA) containing 1 mg PIC (approximately 5 μmol), yielding a final PIC concentration of 200 μg/mL. The suspension was incubated at 37 °C with gentle stirring for 30 min to allow bilayer swelling and drug incorporation. Multilamellar vesicles were then probe-sonicated (SONICS Vibra-Cell, ultrasonic processor, Sonics & Materials, Inc., Newtown, CT, USA) in an ice bath using 10 s pulses at 30% amplitude, intermittently for 10 min. Unencapsulated PIC was removed by centrifugation at 15,000× *g* for 30 min. The supernatant containing PIC-LNPs was characterized for particle size, polydispersity index (PDI), and zeta potential using a Zetasizer Nano ZS (Malvern Instruments, Malvern, UK). Particle size was measured in distilled water, and zeta potential was measured in triplicate. Vesicle morphology and integrity were examined by transmission electron microscopy (TEM; JEM-2100, JEOL Ltd., Tokyo, Japan) at 160 kV.

### 2.3. Encapsulation Efficiency and Drug Loading

Encapsulation efficiency (EE%) and drug loading (DL%) were determined by measuring free (non-encapsulated) PIC in the supernatant. The PIC content was quantified by UV–Vis spectrophotometry (UV–Vis spectrophotometer, model UV-1800, Shimadzu Corporation, Kyoto, Japan) at 260 nm. Encapsulated PIC was calculated by subtracting the free PIC from the total amount added. EE% and DL% were calculated as:

Drug loading (%) = [Encapsulated PIC/Total liposome mass] × 100

Encapsulation efficiency (%) = [Encapsulated PIC/Total PIC added] × 100

### 2.4. Fourier Transform Infrared Spectroscopy (FTIR)

FTIR analysis was used to evaluate functional groups and potential interactions between PIC, lipid components, and PIC-LNPs. Samples were scanned in the range 4000–400 cm^−1^. Characteristic peaks were compared to confirm successful incorporation of PIC into the lipid matrix.

### 2.5. In Vitro Drug Release

The release behaviour of PIC from PIC-LNPs was evaluated in phosphate-buffered saline (PBS, pH 7.4) maintained at 37 °C with mild shaking. At defined time points, samples of the release medium were collected and immediately replaced with fresh PBS. The amount of PIC liberated at each interval was quantified by UV–Vis spectrophotometry at 260 nm, and the percentage of drug released was calculated and plotted over time.

### 2.6. Experimental Animals

Sixty male albino Sprague–Dawley rats (200–300 g) were obtained from the “Medical Experimental Research Center (MERC), Faculty of Medicine, Mansoura University, Mansoura, Egypt.” Animals were clinically inspected, housed in clean plastic cages, and maintained under controlled conditions (25 °C, 45% relative humidity, 12 h light/dark cycle) with free access to standard chow and water. Rats were acclimatized for two weeks before experimentation. All procedures complied with OECD Guideline 420 [22] and were conducted in accordance with internationally accepted standards for animal care and use and aligned with “ARRIVE (Animal Research: Reporting of In Vivo Experiments)” guidelines [23]. The study protocol was approved by the “Research Ethics Committee, Faculty of Veterinary Medicine, Mansoura University, Egypt, approval code MU-ACUC; VM.R.26.03.272.”

### 2.7. Study Design and Treatments

Rats were randomly assigned to six groups (*n* = 10/group):

(1) Control: received vehicle only.
(2) PIC: intraperitoneal (i.p.) PIC (20 mg/kg) once daily for 7 days.
(3) PIC-LNPs: i.p. PIC-LNPs (20 mg/kg equivalent PIC) once daily for 7 days.
(4) MTX: single i.p. MTX dose (20 mg/kg) on day 1.
(5) PIC + MTX: MTX as above, followed by i.p. PIC (20 mg/kg) once daily for 5 days.
(6) PIC-LNPs + MTX: MTX as above, followed by i.p. PIC-LNPs (20 mg/kg equivalent PIC) once daily for 5 days.

This design allowed comparison of free versus liposomal PIC in the context of MTX-induced renal injury. Dose/treatment schedules were adapted from earlier studies and refined through preliminary work to achieve reliable MTX-mediated renal injury, thereby enabling evaluation of the mitigating effects of PIC and PIC-LNPs [3,24,25].

### 2.8. Tissue Collection and Processing

Rats were fasted for 10 h before sacrifice. Anesthesia was induced and maintained with isoflurane, then euthanasia was completed using 5% isoflurane in oxygen for 5 min; the absence of cardiac and respiratory activity confirmed death. In each group, seven rats were used for biochemical and molecular analyses, and three for histopathological and ultrastructural studies. Blood was collected from the retro-orbital plexus, allowed to clot, and centrifuged at 3000× *g* for 10 min. Serum aliquots were stored at −80 °C. Kidneys were removed, rinsed in ice-cold 1.15% KCl, and sectioned; fragments designated for biochemical and molecular work were stored at −20 °C, while others were fixed in 10% neutral buffered formalin for light microscopy and TEM. For biochemical assays, tissue was homogenized in 50 mmol/L Tris–HCl (pH 7.4), centrifuged at 10,000× *g* for 15 min at 4 °C, and the clarified supernatants were preserved at −20 °C pending analysis.

### 2.9. Assessment of Kidney Function

All Kidney function assessment assays were purchased from BioDiagnostic, Cairo, Egypt. Serum creatinine was quantified using a kinetic colorimetric assay based on the Jaffé reaction, with absorbance readings at 495 nm. An enzymatic urease measured serum urea—Berthelot colorimetric method with absorbance recorded at 530–560 nm. Serum uric acid was determined using an enzymatic uricase-peroxidase colorimetric assay, with absorbance measured at 510 nm, according to standard clinical chemistry procedures described in [26].

### 2.10. Antioxidant Defenses and Oxidative Stress Markers

Renal antioxidant status was assessed by measuring reduced glutathione (GSH) and the activities of superoxide dismutase (SOD), glutathione peroxidase (GPx), and catalase (CAT) using validated colorimetric assay kits (Table S1) according to the manufacturers’ instructions [27]. Oxidative damage was evaluated by determining renal reactive oxygen species (ROS), malondialdehyde (MDA), protein carbonyls (PC), and 8-hydroxy-2′-deoxyguanosine (8-OHdG), whereas nuclear factor erythroid 2-related factor 2 (Nrf2) and heme oxygenase-1 (HO-1) levels were quantified using specific ELISA kits (Table S1) [28,29,30].

### 2.11. Inflammatory Mediators and Nitrosative Stress

To evaluate renal inflammation, nuclear NF-κB p65 DNA-binding activity was measured as follows: renal cortical tissue was homogenized in ice-cold lysis buffer, and nuclear proteins were isolated using the Nuclear Extraction Kit (ab113474; Abcam, Cambridge, UK) strictly according to the manufacturer’s instructions. Briefly, cytoplasmic and nuclear fractions were separated by sequential low- and high-speed centrifugation steps as prescribed by the kit protocol, yielding a purified nuclear extract. Nuclear NF-κB p65 DNA-binding activity was subsequently quantified using the NF-κB p65 Transcription Factor Assay Kit (ab133112; Abcam, Cambridge, UK). Fifty micrograms of nuclear extract per well were loaded onto the pre-coated 96-well plate containing an immobilized consensus oligonucleotide (5′-GGGACTTTCC-3′), which selectively captures activated, DNA-binding p65. Absorbance was measured at 450 nm, and results were normalized to total nuclear protein content as determined by the BCA assay. The cytokines TNF-α and IL-1β were measured in total tissue homogenate supernatants using rat-specific ELISA kits, and Toll-like receptor 4 (TLR4) levels were determined with a dedicated ELISA kit for rat kidney tissue (Table S1). Nitrosative stress was assessed by quantifying total nitric oxide (NO) metabolites with a microplate-based colorimetric assay (Table S1). All measurements were carried out according to the manufacturer’s protocols.

### 2.12. Apoptotic Markers

Apoptosis indices in renal tissue were assessed by measuring Bax, Bcl-2, and caspase-3 protein levels. These markers were quantified using rat-specific ELISA kits applied to supernatants from kidney homogenates (Table S1). Assays were conducted strictly according to the manufacturers’ protocols, and the resulting values were normalised to each sample’s total protein content.

### 2.13. Phosphorylated MAPK Proteins

Activation of mitogen-activated protein kinase (MAPK) signalling was investigated by determining phosphorylated ERK1/2, p38 MAPK, and JNK in kidney homogenates. Before analysis, total protein concentrations in the supernatants were established by a BCA assay to allow appropriate normalisation. Phosphorylated forms of ERK1/2, p38, and JNK were then quantified using SimpleStep ELISA kits following the supplier’s instructions (Table S1). Each sample was analysed in duplicate, and absorbance at 450 nm was used to interpolate protein concentrations from standard curves.

### 2.14. RNA Extraction and RT-qPCR

Total RNA was extracted from renal samples using QIAzol Lysis Reagent (Qiagen, Hilden, Germany) according to the manufacturer’s protocol. After phase separation with chloroform, RNA was precipitated using isopropanol, washed with ethanol (both from Sigma-Aldrich, St. Louis, MO, USA), air-dried, and finally dissolved in DNase/RNase-free water. RNA yield and purity were evaluated spectrophotometrically, and equal amounts of RNA were reverse-transcribed into cDNA using the iScript™ cDNA Synthesis Kit (Bio-Rad Laboratories, Hercules, CA, USA). Quantitative real-time PCR was carried out on a Rotor-Gene Q system (Qiagen, Hilden, Germany) with iTaq Universal SYBR Green Supermix (Bio-Rad Laboratories, Hercules, CA, USA) and the gene-specific primers listed in Table 1. Relative transcript levels were calculated by the 2^−ΔΔCt^ method, using β-actin as the internal control [31].

### 2.15. Histopathology

All histopathology-related reagents were purchased from Sigma-Aldrich, St. Louis, MO, USA, except where otherwise specified. Kidney specimens were immersed in 10% neutral buffered formalin (fixative: tissue ≈ 20:; Piochem, Cairo, Egypt) for three days, then processed through graded alcohols, cleared in xylene, and embedded in paraffin wax. Paraffin blocks were sectioned on a rotary microtome, and the sections were rehydrated and stained with hematoxylin and eosin for examination by light microscopy. Tubular and glomerular alterations, inflammation, and hemorrhage were semi-quantitatively scored (0–3) according to AASLD-based criteria (Table 2). For each rat (3 per group), three sections were prepared, and four non-overlapping fields per section (400×) were examined (12 fields/rat); mean scores were calculated per animal.

### 2.16. Transmission Electron Microscope

All related reagents were obtained from Sigma-Aldrich (St. Louis, MO, USA). For ultrastructural evaluation, kidney pieces were initially fixed in 2.5% glutaraldehyde prepared in 0.1 M phosphate buffer (pH 7.4) at 4 °C for 24 h, rinsed in the same buffer, and then post-fixed in 1% osmium tetroxide for 2 h. Specimens were subsequently dehydrated through ascending ethanol concentrations, passed through acetone, and embedded in epoxy resin. Ultrathin sections (approximately 60–70 nm) were cut, collected on copper grids, and contrasted with uranyl acetate followed by lead citrate before examination in a JEM-2100 transmission electron microscope (JEOL Ltd., Tokyo, Japan) operating at 160 kV.

### 2.17. NRF2 and NF-κB Immunohistochemical Analysis

Paraffin-embedded renal sections were deparaffinised in xylene, rehydrated through graded ethanols, and subjected to heat-induced antigen retrieval in 0.01 M citrate buffer (pH 6.0). Endogenous peroxidase activity was quenched with 3% H_2_O_2_ in methanol, and non-specific binding was minimised by pre-incubation with 5% bovine serum albumin (all from Sigma-Aldrich, St. Louis, MO, USA). Sections were then incubated overnight at 4 °C with rabbit anti-Nrf2 and anti-NF-κB p65 primary antibodies (Abcam, Cambridge, UK; 1:200). After PBS washing, a biotinylated goat anti-rabbit secondary antibody, followed by streptavidin–HRP, was applied sequentially according to the supplier’s recommendations. Immunoreactivity was visualized with DAB and counterstained with Mayer’s hematoxylin, then dehydrated, cleared, and mounted with DPX. Stained sections were examined at 400× magnification. Five random fields per section were photographed, and the percentage of positively stained area was quantified using Fiji (ImageJ, version 1.54g; National Institutes of Health, Bethesda, MD, USA).

### 2.18. Blinding and Statistical Analysis

The investigators responsible for drug administration and sample collection were aware of the group allocation. Histopathological and immunohistochemical evaluations were performed by an experienced pathologist who was blinded to the treatment groups; kidney sections were coded before scoring and image analysis. Biochemical, ELISA, and RT-qPCR assays were carried out according to predefined protocols using coded samples, and the operator was not informed of group identity during measurement. Statistical analyses were conducted on datasets labeled only with group codes, and the treatment allocation was revealed only after completion of the primary analyses.

Before analysis, datasets were examined for normal distribution and equality of variances using the Shapiro–Wilk and Levene tests, respectively. Group comparisons were then performed using one-way analysis of variance (ANOVA) in SAS (version 2012; PROC ANOVA). When a significant overall effect was detected, Tukey’s honestly significant difference test was applied for pairwise post hoc comparisons. Data are presented as mean ± standard error (SE), and differences were regarded as statistically significant when *p* < 0.05. All graphical summaries were created with GraphPad Prism (version 9.5.1, GraphPad Software, San Diego, CA, USA).

## 3. Results

### 3.1. Physicochemical Characterization of PIC-LNPs

**Figure 2:**
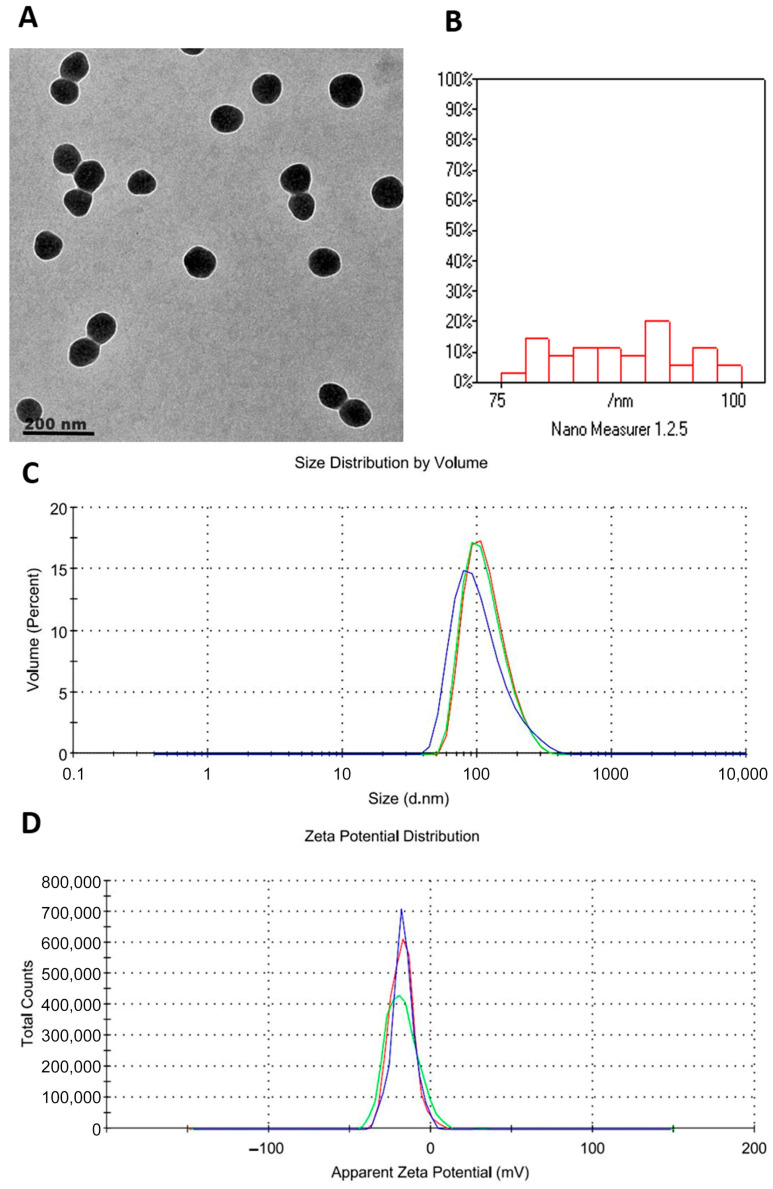
Physicochemical Characterization of PIC-LNPs: (**A**) TEM Micrograph Showing Predominantly Spherical Morphology, (**B**) Particle Size Distribution Histogram Indicating Most Particles Range Between 75 and 100 nm with Narrow Dispersion, (**C**) Zeta Size Distribution by Volume, and (**D**) Zeta Potential Profile.

Figure 2A shows PIC-LNPs with clear spherical shapes and no aggregation. This confirms their structural integrity. Particle size measurements (Figure 2B) ranged from 75 to 100 nm. The results indicate uniform and consistent distribution. DLS analysis (Figure 2C) indicated that PIC-LNPs possess an average hydrodynamic diameter of 128 nm and a low PDI of 0.157, confirming their consistent and narrowly distributed particle sizes. The zeta potential of PIC-LNPs was measured at −37 mV (Figure 2D), suggesting excellent colloidal stability.

### 3.2. Encapsulation Efficiency and Drug Loading Results

The PIC-LNPs showed high encapsulation efficiency (~90%) and drug loading (~5.5%), demonstrating effective incorporation of Piceatannol into the lipid matrix. These values confirm the formulation’s ability to deliver the drug efficiently while maintaining stability.

### 3.3. Fourier Transform Infrared Spectroscopy (FTIR) Analysis

**Figure 3:**
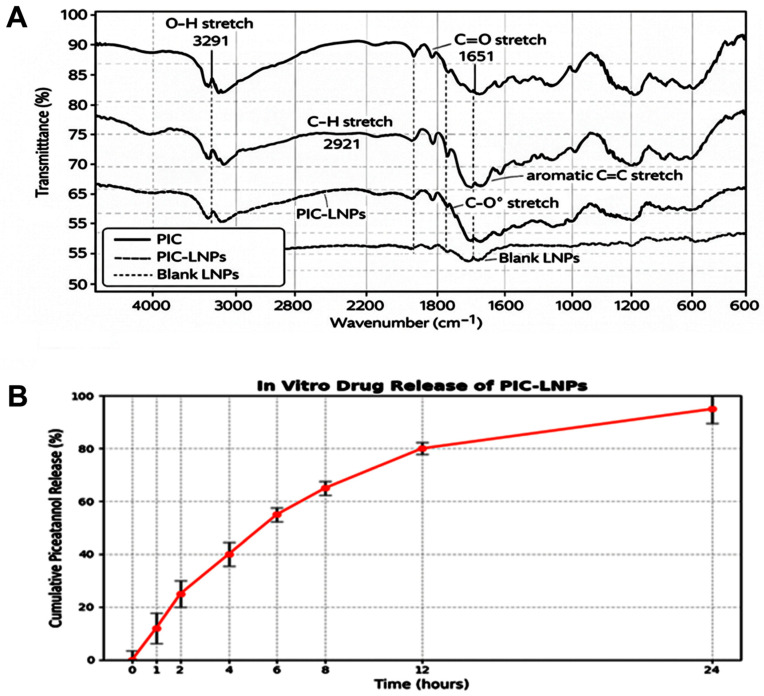
(**A**) FTIR spectra of Piceatannol (PIC), PIC-loaded liposomal nanoparticles (PIC-LNPs), and blank liposomes (Blank LNPs), highlighting key functional group peaks and confirming successful drug incorporation. (**B**) Cumulative in vitro release of Piceatannol from PIC-LNPs over 24 h, showing gradual drug release with variable standard deviations at each time point.

The FTIR spectra (Figure 3A) reveal clear interactions between Piceatannol (PIC) and the liposomal matrix. PIC shows characteristic O–H stretching around 3290 cm^−1^, C–H stretching near 2920 cm^−1^, and C=O stretching at 1650 cm^−1^. In the PIC-loaded liposomes (PIC-LNPs), these peaks are slightly shifted and broadened, indicating successful incorporation of PIC into the lipid bilayers without forming new chemical bonds. The blank liposomes show only typical lipid peaks, confirming that the observed changes in PIC-LNPs arise from drug encapsulation.

### 3.4. In Vitro Drug Release Results

The release profile of Piceatannol from the liposomal nanoparticles (PIC-LNPs) shows a gradual and sustained pattern over 24 h (Figure 3B). Around 12% of the drug was released within the first hour, followed by a steady increase to nearly 80–95% by 24 h. This sustained release highlights the liposomal matrix’s ability to deliver drugs in a controlled manner over an extended period.

### 3.5. Effect of PIC Formulations on Renal Function Biomarkers

**Figure 4:**
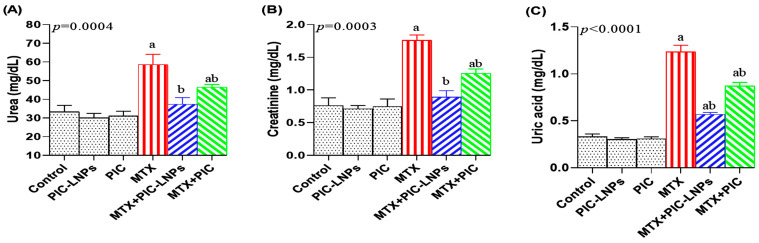
Effect of Crude and Nanoliposomal PIC on Serum Renal Function Biomarkers in Methotrexate-Induced Nephrotoxicity: (**A**) Urea, (**B**) Creatinine, and (**C**) Uric Acid Compared with the Normal Control Group; Values are reported as mean ± SE. Marks “a” and “b” indicate statistically significant differences from the control and MTX-treated groups, respectively.

The administration of MTX resulted in a marked deterioration of renal function, as evidenced by significant elevations in serum urea by 76.24% (Figure 4A), creatinine by 141.56% (Figure 4B), and uric acid levels by 278.29% (Figure 4C) compared with the normal control group. Treatment with PIC, in both its crude and nanoliposomal formulations, significantly reduced the levels of these renal biomarkers. Notably, the nanoliposomal PIC formulation demonstrated greater efficacy than the crude form. However, no significant differences were detected between the MTX-exposed group and the MTX + PIC-LNPs group regarding serum urea and creatinine levels.

### 3.6. Effect of PIC Formulations on Renal Nrf2/HO-1 Signaling, Antioxidant Defenses, and Oxidative/Nitrosative Stress

**Figure 5:**
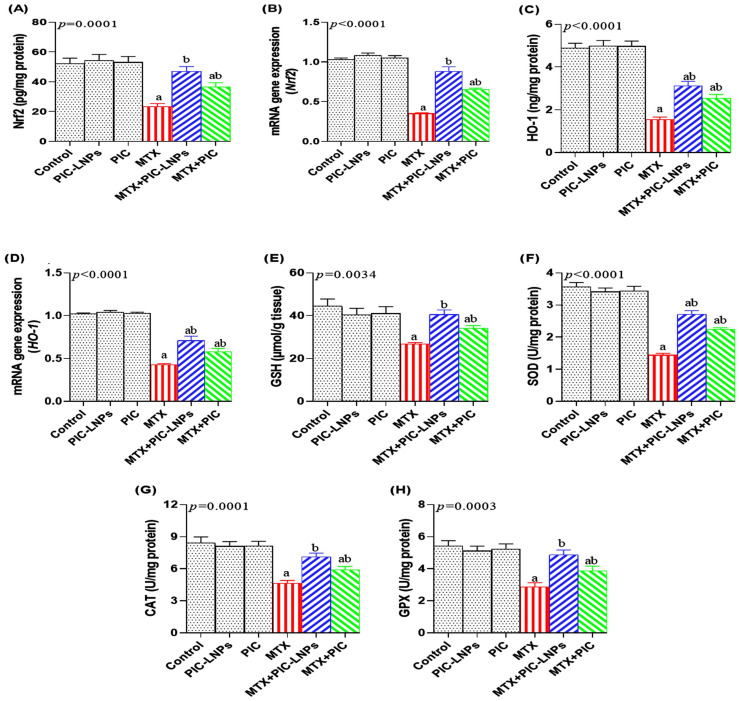
Effect of crude and nanoliposomal piceatannol on renal Nrf2/HO-1 signaling and antioxidant status in methotrexate-induced nephrotoxicity. (**A**,**B**) Nrf2 protein and mRNA expression; (**C**,**D**) HO-1 protein and mRNA expression; (**E**) GSH content; (**F**) SOD activity; (**G**) CAT activity; (**H**) GPx activity. Data are presented as mean ± SE. Letters “a” and “b” denote significant differences versus the control and MTX groups, respectively (*p* < 0.05).

MTX administration led to a clear downregulation of the renal Nrf2/HO-1 axis, as reflected by significant decreases in Nrf2 protein and transcript levels by nearly 55.60% (Figure 5A,B) and in HO-1 protein and transcript levels by 68.53% (Figure 5C,D). In contrast, co-treatment with PIC, particularly in its liposomal form, counteracted this suppression. In the MTX + PIC-LNPs group, Nrf2 expression at both the protein and gene levels was restored to values that did not differ from the control group. In parallel, MTX markedly depleted renal GSH content by 39.00% (Figure 5E) and significantly reduced the activities of SOD by 59.55% (Figure 5F), CAT by 44.84% (Figure 5G), and GPx by 47.32% (Figure 5H). Both PIC preparations improved these antioxidant parameters, with the PIC-LNPs regimen producing the most pronounced recovery. Notably, GSH, CAT, and GPx values in MTX + PIC-LNPs rats were comparable to those of the normal controls.

**Figure 6:**
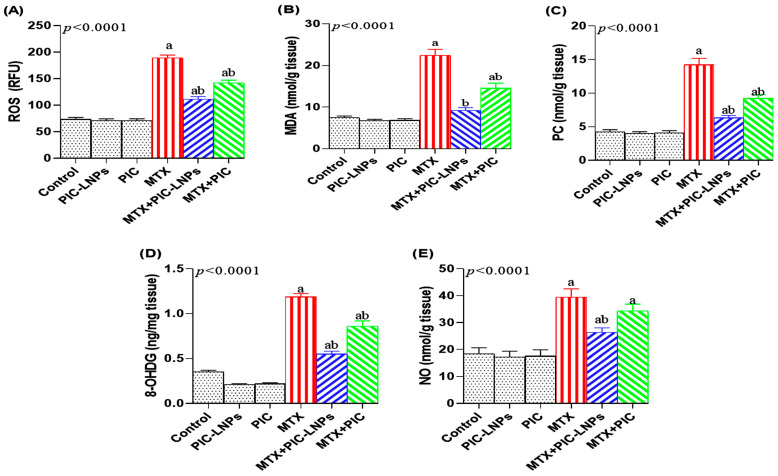
Effect of crude and nanoliposomal piceatannol on renal oxidative and nitrosative stress markers in methotrexate-induced nephrotoxicity. (**A**) ROS; (**B**) MDA; (**C**) protein carbonyls (PC); (**D**) 8-OHdG; (**E**) nitric oxide (NO). Data are presented as mean ± SE. Letters “a” and “b” denote significant differences versus the control and MTX groups, respectively (*p* < 0.05).

Consistent with impaired antioxidant status, MTX exposure substantially increased indices of oxidative damage, including renal ROS by 158.01% (Figure 6A), MDA by 201.68% (Figure 6B), protein carbonyls by 237.93% (PC; Figure 6C), and the DNA oxidation marker 8-OHdG by 241.96% (Figure 6D), relative to the negative control group. Co-administration of PIC in either form significantly attenuated these changes, with the nanoliposomal formulation again providing superior protection. In the MTX + PIC-LNPs group, MDA levels were reduced to values indistinguishable from those of the control rats. Nitrosative stress followed a similar pattern: renal NO (Figure 6E) was significantly elevated after MTX treatment by 117.75%, and this increase was effectively blunted only in the PIC-LNPs group. In contrast, crude PIC did not significantly modify NO levels compared with MTX alone.

### 3.7. Effect of PIC Formulations on Renal TLR4/NF-κB Signaling and Pro-Inflammatory Cytokines

**Figure 7:**
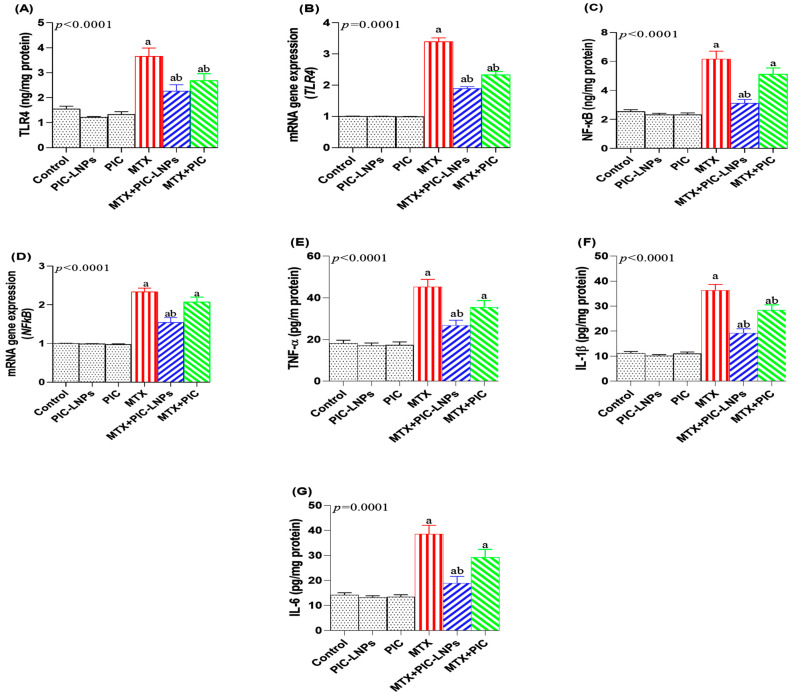
Effect of crude and nanoliposomal piceatannol on renal TLR4/NF-κB signaling and pro-inflammatory cytokines levels in methotrexate-induced nephrotoxicity. (**A**,**B**) TLR4 protein and mRNA expression; (**C**,**D**) NF-κB protein and mRNA expression; (**E**) TNF-α; (**F**) IL-1β; (**G**) IL-6. Data are presented as mean ± SE. Letters “a” and “b” denote significant differences versus the control and MTX groups, respectively (*p* < 0.05).

In MTX-treated rats, the renal TLR4/NF-κB pathway was markedly activated, as indicated by significant elevations in TLR4 protein and gene expression by 134.73% (Figure 7A,B) and in NF-κB protein and gene expression by 132.62% (Figure 7C,D) relative to controls. Co-treatment with PIC attenuated this activation, with the liposomal formulation exerting a more robust effect than the crude compound. Of note, nuclear NF-κB p65 was measured in purified nuclear fractions isolated from renal cortical homogenates, thereby specifically reflecting activated, nuclear-translocated p65 rather than total cellular p65 protein. The NF-κB levels in the MTX + crude PIC group did not differ significantly from those in MTX-only animals at either the protein or mRNA level, underscoring the limited impact of the non-encapsulated form on this pathway.

MTX exposure also resulted in a significant rise in renal pro-inflammatory cytokines TNF-α by 150.51% (Figure 7E), IL-1β by 223.36% (Figure 7F), and IL-6 by 169.99% (Figure 7G) compared with the negative control group. Co-administration of PIC-LNPs with MTX significantly lowered these cytokine levels and outperformed crude PIC. For TNF-α and IL-6, values in the MTX + crude PIC group remained comparable to those in the MTX-only group, whereas PIC-LNPs produced a clear anti-inflammatory effect.

### 3.8. Effect of PIC Formulations on Renal MAPK Signaling

**Figure 8:**
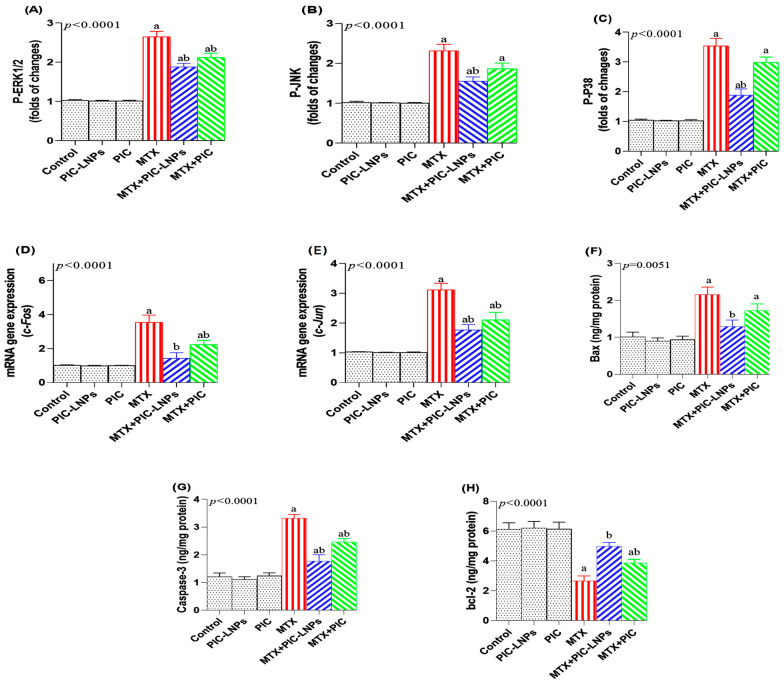
Effect of crude and nanoliposomal piceatannol on renal MAPK signaling and apoptosis markers in methotrexate-induced nephrotoxicity. (**A**) p-ERK1/2; (**B**) p-JNK; (**C**) p-p38; (**D**) c-Fos transcript; (**E**) c-Jun transcript; (**F**) Bax; (**G**) caspase-3; (**H**) Bcl-2. Data are presented as mean ± SE. Letters “a” and “b” denote significant differences versus the control and MTX groups, respectively (*p* < 0.05).

MTX challenge activated the renal MAPK cascade. Relative renal protein expression analysis demonstrated that phosphorylated ERK1/2 increased by 156.94% (p-ERK1/2; Figure 8A), phosphorylated JNK increased by 126.92% (p-JNK; Figure 8B), and phosphorylated p38 increased by 239.53% (p-p38; Figure 8C) compared with the normal control group, confirming marked activation of MAPK phosphorylation. In parallel, the downstream transcription factors c-Fos and c-Jun were significantly elevated by 245.67% (Figure 8D) and 202.55% (Figure 8E), respectively. Co-treatment with PIC-LNPs significantly dampened this activation. In the MTX + PIC-LNPs group, phosphorylation levels of ERK1/2, JNK, and p38 were markedly reduced compared with the MTX group, accompanied by suppression of downstream c-Fos and c-Jun expression. In rats receiving MTX + PIC-LNPs, c-Fos expression was normalized to values that were not significantly different from those of controls. By contrast, p-JNK and p-p38 levels in the MTX + crude PIC group remained similar to those of the MTX-only group, indicating that the crude formulation failed to restrain these MAPKs effectively.

### 3.9. Effect of PIC Formulations on Apoptotic Markers

MTX exposure markedly enhanced renal apoptosis, as evidenced by significant increases in the pro-apoptotic proteins Bax (77.05%; Figure 8F) and caspase-3 (169.20%; Figure 8G) compared with the control group. Co-administration of PIC-LNPs substantially reduced the levels of both markers, whereas crude PIC had a more modest effect. In fact, Bax concentrations in the MTX + crude PIC group did not differ significantly from those in MTX-only rats. Conversely, MTX significantly decreased the anti-apoptotic protein Bcl-2 by 56.86% (Figure 8H). Treatment with PIC-LNPs effectively restored Bcl-2 to near-normal levels, with no significant difference versus the control group, highlighting a more favorable Bax/Bcl-2 balance in the liposomal PIC-treated animals.

### 3.10. Histopathological Findings

**Figure 9:**
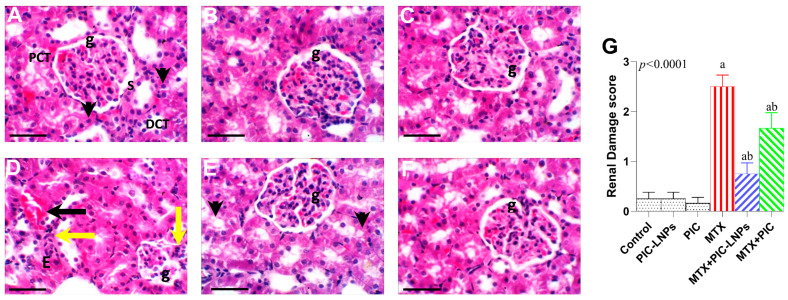
Representative photomicrographs of renal histology in control and treated rats. (**A**) Control; (**B**) PIC-LNPs; (**C**) PIC; (**D**) MTX; (**E**) MTX + PIC; (**F**) MTX + PIC-LNPs. Photomicrographs of kidney sections from (**A**–**C**) groups show well-preserved renal architecture. The glomeruli (g) appear intact with normal Bowman’s spaces (S), and both proximal (PCT) and distal (DCT) convoluted tubules are clearly organized. The tubular epithelial cells (arrowheads) retain normal cytoplasmic features and distinct nuclei, indicating healthy tissue structure. In contrast, the (**D**) MTX group displays clear histopathological damage, including distortion of the glomerular tuft (g), vascular congestion (black arrow), and noticeable degeneration of the tubular epithelial cells. These changes are accompanied by interstitial edema “E” and lymphocytic infiltration around the glomeruli and renal tubules (yellow arrows). Meanwhile, the (**E**) MTX + PIC-LNPs and (**F**) MTX + PIC-LNPs treated groups show a marked improvement, with kidney architecture largely restored. The glomeruli (g) regain their normal structure, and only mild vacuolation is observed in the tubular epithelial cells (arrowheads). H&E, 400×; scale bar = 50 µm. (**G**) Semi-quantitative renal damage scores in control and treated groups. Data are presented as mean ± SE. Letters “a” and “b” denote significant differences versus the control and MTX groups, respectively (*p* < 0.05).

Light microscopic examination of kidney sections from the negative control, PIC-LNPs, and PIC groups showed preserved renal architecture, with normal-appearing glomeruli and intact proximal and distal tubules (Figure 9A–C). In contrast, MTX-treated rats exhibited extensive structural damage, including glomerular atrophy, marked vascular congestion, and prominent periglomerular lymphocytic infiltration (Figure 9D). Co-treatment with either crude PIC or PIC-LNPs mitigated these lesions. Renal sections from the MTX + PIC and MTX + PIC-LNPs groups showed largely preserved glomerular and tubular structures with only mild congestion (Figure 9E,F). Semi-quantitative scoring confirmed a significant increase in renal injury in the MTX group compared with controls and antioxidant-only groups, partial improvement with crude PIC, and the greatest reduction in damage scores in the PIC-LNPs–treated animals (Figure 9G).

### 3.11. Ultrastructural Findings

**Figure 10:**
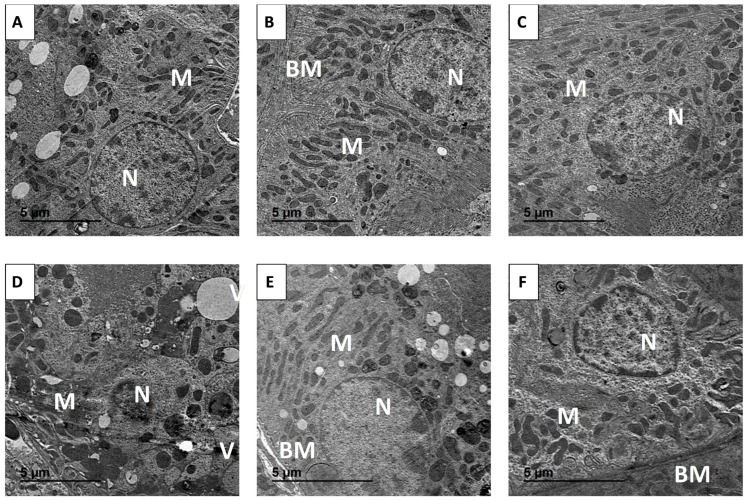
Transmission electron micrographs of renal cortex in control and treated groups. (**A**) Control; (**B**) PIC; (**C**) PIC-LNPs; (**D**) MTX; (**E**) MTX + PIC; (**F**) MTX + PIC-LNPs. Nucleus (N), mitochondria (M), cytoplasmic vacuolation (V), and basement membrane (BM) are indicated.

Electron microscopy of proximal tubular epithelial cells from control, PIC-LNPs, and PIC groups revealed normal ultrastructure, characterized by an intact basement membrane, dense apical brush border, rounded euchromatic nuclei, and abundant elongated mitochondria (Figure 10A–C). MTX exposure produced severe ultrastructural alterations, including shrunken, heterochromatic nuclei, extensive basal cytoplasmic vacuolation, swollen and fragmented mitochondria, and shortened, irregular apical microvilli (Figure 10D). Co-treatment with PIC-LNPs or crude PIC markedly improved these features. In the MTX + PIC and MTX + PIC-LNPs groups, proximal tubular cells showed largely restored mitochondrial morphology, euchromatic nuclei, and reformed brush borders, with substantially fewer vacuoles compared with the MTX-only group (Figure 10E,F).

### 3.12. Immuno-Histochemistry Findings

**Figure 11:**
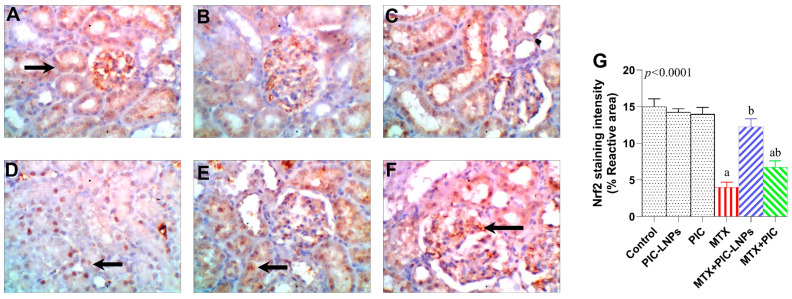
Representative photomicrographs of Nrf2 immunolocalization in the rat kidney. (**A**–**C**) Control groups showing strong Nrf2 immunoreactivity in cortical tubules; (**D**) MTX group with weak Nrf2 staining (arrows); (**E**) MTX + PIC-LNPs and (**F**) MTX + PIC groups showing marked enhancement of Nrf2 immunostaining. Original magnification 400×. (**G**) Quantitative analysis of Nrf2 immunostaining intensity in renal cortex across experimental groups. Data are presented as mean ± SE. Letters “a” and “b” denote significant differences versus the control and MTX groups, respectively (*p* < 0.05).

In kidneys from control animals, Nrf2 immunoreactivity was intense and diffusely distributed in cortical tubular epithelial cells, consistent with an active endogenous antioxidant defense (Figure 11A–C). MTX treatment markedly diminished Nrf2 staining, which became weak and patchy within the tubular epithelium (Figure 11D). PIC co-treatment partially restored Nrf2 positivity (Figure 11E), whereas PIC-LNPs produced strong and widespread staining throughout the cortical tubules (Figure 11F). Quantitative analysis confirmed a significant MTX-induced reduction in Nrf2 immunostaining intensity and a graded restoration with PIC and PIC-LNPs, with the highest values observed in the MTX + PIC-LNPs group (Figure 11G).

### 3.13. NF-κB Immunohistochemistry

**Figure 12:**
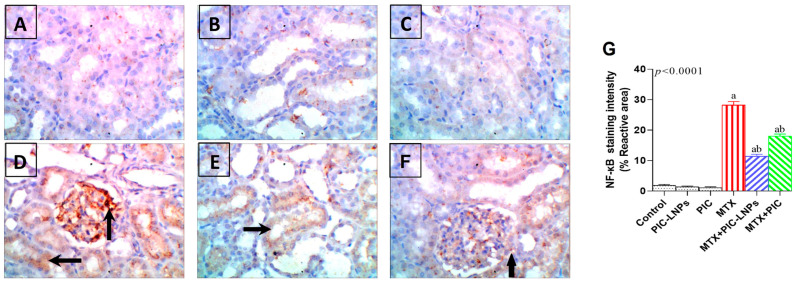
Representative photomicrographs of NF-κB immunoreactivity in the rat kidney. (**A**–**C**) Control groups with minimal NF-κB staining; (**D**) MTX group showing strong NF-κB immunolocalization in proximal tubular epithelial cells around glomeruli (arrows); (**E**,**F**) MTX + PIC-LNPs and MTX + PIC groups exhibiting weak NF-κB staining in cortical tubules, approaching control patterns. Original magnification 400×. (**G**) Quantitative analysis of NF-κB immunostaining intensity in renal cortex across experimental groups. Data are presented as mean ± SE. Letters “a” and “b” denote significant differences versus the control and MTX groups, respectively (*p* < 0.05).

NF-κB expression was barely detectable in renal cortical tubules of control rats, indicating a basal, non-activated inflammatory status (Figure 12A–C). MTX exposure elicited a marked increase in NF-κB immunostaining, particularly in proximal tubular epithelial cells surrounding the glomeruli, with intense brown nuclear and cytoplasmic labeling (Figure 12D). Treatment with PIC or PIC-LNPs clearly attenuated this response. In the MTX + PIC and MTX + PIC-LNPs groups, NF-κB staining in cortical tubules was weak and approached control levels, with the most pronounced reduction observed in the nanoparticle-treated animals (Figure 12E,F). Densitometric analysis corroborated these observations: MTX significantly elevated NF-κB immunostaining, and both PIC formulations reduced this increase, with PIC-LNPs achieving the greatest normalization (Figure 12G).

## 4. Discussion

The present data show that MTX profoundly compromises renal function, as reflected by significant rises in serum urea and creatinine, two global indicators of glomerular filtration efficiency [32]. Their sustained elevation in the MTX group confirms clinically relevant nephrotoxicity and impaired kidney performance, in agreement with previous reports of MTX-induced renal injury [5,33]. Mechanistically, MTX and its metabolites can precipitate and crystallize within tubular lumina, leading to intratubular obstruction and direct epithelial injury [3]. In parallel, MTX-driven oxidative and inflammatory responses further deteriorate glomerular hemodynamics and filtration capacity [34]. The concomitant increase in serum uric acid in MTX-treated rats likely reflects accelerated purine catabolism and impaired renal clearance, which favor intratubular crystal deposition and aggravate oxidative burden [35]. Similar alterations have been linked with delayed MTX elimination and evolving renal dysfunction [36]. Ultrastructural analyses in the current work corroborated these biochemical changes by revealing clear structural damage to nephron segments. Co-treatment with PIC-LNPs markedly lowered serum urea, creatinine, and uric acid compared with MTX alone and exceeded the renoprotective effect of crude PIC, indicating more effective preservation of both renal function and microstructure. This superiority is plausibly related to enhanced bioavailability and sustained tissue exposure achieved through liposomal encapsulation [37]. Our findings are consistent with prior studies showing that antioxidant polyphenols such as pycnogenol mitigate MTX-induced renal impairment in rats [6], and with reports that resveratrol, epigallocatechin gallate, and other polyphenols ameliorate urea and creatinine elevations in diverse experimental nephrotoxicity models [38,39], further supporting polyphenol-based strategies for limiting MTX-associated kidney injury.

In the current study, MTX produced a profound disturbance in renal redox balance, underscoring its ability to trigger both oxidative and nitrosative stress [38,40]. This imbalance was evidenced by reduced GSH levels and depressed activities of GPx, CAT, and SOD, together with robust increases in MDA, ROS, protein carbonyls, and 8-OHdG, reflecting extensive lipid, protein, and DNA oxidation. Such redox derangement is likely driven by mitochondrial dysfunction and by the upregulation of ROS-generating systems, such as NADPH oxidase, which collectively promote sustained ROS overproduction and the exhaustion of endogenous defenses [41]. The concomitant rise in NO points to the induction of iNOS and formation of peroxynitrite, thereby amplifying nitrosative stress and contributing to macromolecular injury [42]. MTX also significantly downregulated Nrf2 and HO-1, two central components of the antioxidant response pathway [43]. Attenuation of this signaling axis reduces the transcription of detoxifying and antioxidant genes, leaving renal cells highly vulnerable to ROS/RNS-mediated damage. In parallel, GSH depletion narrows the buffering capacity against hydroxyl radical, superoxide, and hydrogen peroxide, further sensitizing renal tissue to oxidative attack [44]. Our observations of increased MDA and reduced SOD, CAT, and GPx activities mirror earlier descriptions of oxidative injury in MTX nephrotoxicity [3,44,45]. Importantly, these findings support a mechanistic link between oxidative stress and downstream inflammatory and apoptotic signaling. The observed downregulation of Nrf2 not only diminishes antioxidant defense but may also indirectly facilitate NF-κB activation, creating crosstalk between oxidative and inflammatory pathways. Therefore, MTX-induced renal injury cannot be considered exclusively as oxidative damage but rather as a coordinated disruption of multiple interdependent cellular signaling networks. Notably, PIC-LNPs administration in the present model significantly restored GSH content and antioxidant enzyme activities, reduced markers of oxidative damage. It upregulated Nrf2 and HO-1 expression, indicating a broad reinforcement of the endogenous antioxidant network. These outcomes are in line with the known radical-scavenging, metal-chelating, and Nrf2-activating properties of piceatannol [46]. By concomitantly attenuating NO levels, PIC-LNPs may also limit peroxynitrite generation and nitrosative damage in renal tissue. Similar improvements in GSH, enzymatic antioxidants, and lipid peroxidation after polyphenol treatment have been reported in MTX-exposed animals [6], supporting the concept that nanoformulated piceatannol can counter both oxidative and nitrosative stress components induced by MTX.

MTX-related renal inflammation is closely linked to activation of the TLR4/NF-κB signaling cascade [47]. Cellular stress and release of damage-associated molecular patterns in response to MTX can engage TLR4, a key pattern-recognition receptor for tissue injury [48]. Upon activation, TLR4 recruits adaptor proteins such as MyD88, initiating downstream signaling that culminates in NF-κB activation and nuclear translocation [49]. Active NF-κB binds to promoter regions of inflammatory genes, driving increased expression of TNF-α, IL-6, and IL-1β [50]. Each cytokine contributes distinct but complementary effects: TNF-α disrupts endothelial barrier integrity and enhances leukocyte adhesion and transmigration, IL-6 coordinates acute-phase responses and systemic inflammation, and IL-1β intensifies local inflammatory signaling and vascular activation [51]. Together, these mediators create a self-sustaining inflammatory loop that exacerbates tubular and interstitial injury and fuels additional ROS production. In this context, PIC-LNPs in our study produced a marked attenuation of TLR4/NF-κB activation and a substantial decline in TNF-α, IL-6, and IL-1β levels, with an effect stronger than that of free PIC. These findings are consistent with previous work demonstrating that piceatannol downregulates TLR4 expression and inhibits NF-κB activation by blocking IκB phosphorylation and stabilizing the IκB–NF-κB complex, thereby preventing its nuclear translocation [52,53]. This dual targeting of upstream receptor signaling and downstream transcriptional activation highlights the precision with which piceatannol-containing formulations can modulate MTX-induced renal inflammation.

Crucially, PIC-LNPs mitigated NF-κB activation, not only through direct inhibition but also by indirectly restoring Nrf2-dependent antioxidant defenses. This integrated mechanism highlights that PIC-LNPs act through both suppression of pro-inflammatory transcriptional programs and enhancement of endogenous cytoprotective pathways, demonstrating a multi-targeted mode of action rather than a purely antioxidant effect.

The pro-apoptotic profile observed in MTX-treated rats further illustrates its detrimental effect on renal cell survival. Our results showed increased Bax and caspase-3, together with decreased Bcl-2, culminating in an elevated Bax/Bcl-2 ratio, a hallmark of intrinsic mitochondrial apoptosis [54,55]. Elevated ROS and high levels of pro-inflammatory cytokines, particularly TNF-α, may destabilize mitochondrial membrane potential, alter ATP production, and favor the release of cytochrome c into the cytosol [56]. This event activates downstream caspases, including caspase-3, driving tubular epithelial cell loss and functional decline [57]. Our findings parallel those of Fahmy et al. [25], who reported Bax upregulation, caspase-3 activation, and Bcl-2 suppression in MTX-induced nephrotoxicity. In contrast, PIC-LNPs treatment in the present study normalized Bcl-2 expression while downregulating Bax and caspase-3, effectively correcting the Bax/Bcl-2 ratio and limiting activation of the mitochondrial apoptotic cascade. These results concur with prior evidence that piceatannol preserves mitochondrial membrane potential and reduces caspase-mediated apoptosis under oxidative conditions [58], and with data showing that paeonol and other polyphenols protect against MTX-induced renal apoptosis by modulating mitochondrial pathways [54]. These effects are likely downstream consequences of combined antioxidant and anti-inflammatory mechanisms, linking ROS and NF-κB modulation to apoptosis regulation.

**Figure 13:**
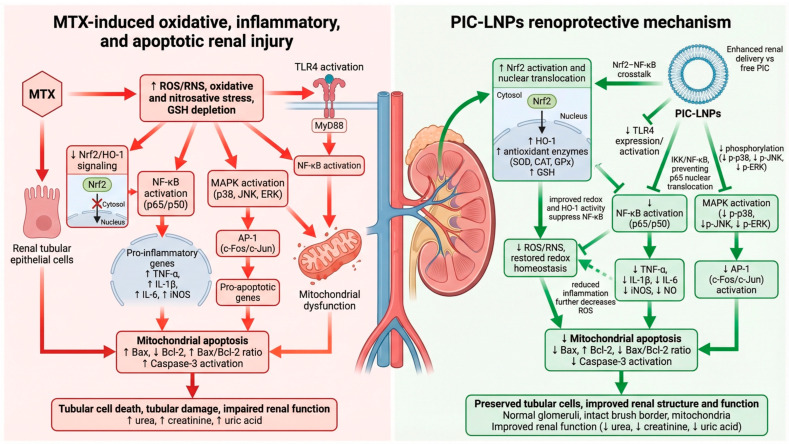
Proposed mechanism of PIC-LNP–mediated protection against MTX-induced renal injury. MTX administration triggers excessive ROS/RNS generation, GSH depletion, and suppression of Nrf2/HO-1 signaling, leading to TLR4 activation, NF-κB and MAPK (p38, JNK, ERK) stimulation, upregulation of pro-inflammatory cytokines and iNOS, and activation of the intrinsic mitochondrial apoptotic pathway (↑ Bax, ↑ caspase-3, ↓ Bcl-2), culminating in tubular damage and renal dysfunction. PIC-LNPs enhance Nrf2/HO-1-dependent antioxidant defenses, restore redox homeostasis, and concurrently suppress TLR4/NF-κB and MAPK signaling, thereby reducing cytokine and iNOS production, limiting mitochondrial apoptosis, and preserving renal structure/function.

Beyond its direct impact on mitochondrial apoptosis, MTX also activates stress-activated kinase pathways that integrate oxidative cues with inflammatory and death signaling. In our model, MTX strongly stimulated renal MAPK signaling, as reflected by increased phosphorylation of p38, JNK, and ERK. These kinases are known to transduce oxidative and inflammatory signals to nuclear effectors, amplifying pro-inflammatory gene transcription and promoting apoptotic responses [11,59]. MAPK activation can function upstream of both NF-κB and mitochondrial apoptosis, thereby intensifying cytokine production and caspase-dependent cell death [50]. Notably, PIC-LNPs in the current study markedly reduced phosphorylation of MAPK components and normalized downstream transcription factors such as c-Fos and c-Jun, indicating effective interruption of stress-activated kinase cascades. This observation further reinforces a mechanistic network in which PIC-LNPs simultaneously attenuate oxidative stress, NF-κB-mediated inflammation, and MAPK-driven apoptosis, providing a unified explanation for their renoprotective effects. Taken together, our data indicate that PIC-LNPs exert renoprotection through an integrated, multitarget mechanism in which enhanced Nrf2/HO-1-dependent antioxidant defenses are coupled with suppression of TLR4/NF-κB and MAPK signaling. This integrated mechanism is summarized schematically in Figure 13, which illustrates how PIC-LNP–induced activation of Nrf2/HO-1 intersects with inhibition of TLR4/NF-κB and MAPK signaling to limit oxidative, inflammatory, and apoptotic injury. By restoring Nrf2 signaling, PIC-LNPs improve redox homeostasis and limit ROS/RNS generation, which attenuates redox-sensitive TLR4/NF-κB activation and pro-inflammatory cytokine production. In parallel, direct inhibition of NF-κB and downregulation of MAPKs further dampen inflammatory amplification of oxidative stress and mitochondrial apoptosis. This network-level modulation is consistent with reports that piceatannol activates Nrf2/HO-1 while directly interfering with IKK/NF-κB and MAPK pathways in nephrotoxic and inflammatory settings, supporting the view that PIC-LNPs act as pleiotropic cytoprotective agents rather than simple radical scavengers [60,61,62,63,64]. Notably, the absence of statistically significant changes in oxidative stress markers, inflammatory mediators, and apoptotic indices in PIC-LNP-treated non-challenged control animals (Groups 2 and 3) is consistent with the stress-context-dependent pharmacology of polyphenolic antioxidants. Under physiological baseline conditions, the measured mediators operate within their homeostatic range, and endogenous antioxidant defenses are not maximally recruited [65,66]; consequently, the capacity of PIC to modulate Nrf2/HO-1 signaling, suppress NF-κB/TLR4 activation, and limit apoptotic progression remains latent unless an oxidative challenge is present [67,68]. This dose-dependent, stimulus-gated response pattern has been documented for structurally related stilbenes and flavonoids, wherein cytoprotective pathway activation requires a threshold level of reactive oxygen species or pro-inflammatory signaling to become fully engaged [69,70].

Collectively, the biochemical findings align well with the histological and ultrastructural observations, in which PIC-LNPs-treated rats showed better preserved glomerular and tubular architecture, fewer degenerative changes, and more intact mitochondria and brush borders. These converging lines of evidence support the concept that nanoformulated piceatannol confers integrated protection by simultaneously targeting oxidative stress, inflammation, and apoptosis.

Some limitations of the present work should be acknowledged. First, the study was conducted in male Sprague–Dawley rats using an acute MTX-induced nephrotoxicity model, which may not fully capture the complexity and chronicity of MTX exposure or sex-related differences in humans. Second, the sample size, while sufficient to detect statistically significant differences in the primary biochemical and histological endpoints, was relatively modest and may limit the precision of effect size estimates and the detection of subtler intergroup differences. Third, mechanistic conclusions are based on a selected panel of oxidative stress, inflammatory, MAPK, and apoptotic markers; additional omics-based or long-term functional studies would be valuable to confirm pathway involvement and assess the durability of the observed protective effects. Fourth, although Nrf2, NF-κB, and MAPK pathway components were assessed using RT-PCR, ELISA, and immunohistochemical analyses (For Nrf2 and NF-κB), Western blot analysis was not available to determine total and phosphorylated protein expression ratios; therefore, additional immunoblot-based confirmation would further strengthen the mechanistic interpretation of these signaling pathways. Finally, the immunohistochemical detection of Nrf2 in renal tissue provided qualitative localization; however, the diffuse signal limits precise nuclear visualization. RT-PCR and ELISA data consistently confirm Nrf2 upregulation, supporting the observed antioxidant response. Future investigations employing high-resolution imaging or nuclear fraction Western blotting will improve mechanistic comprehension. Also, it should be noted that immunohistochemical analysis of NF-κB provides limited insights due to its complex regulation; nonetheless, pathway activation was confirmed by increased TLR4 expression and higher levels of pro-inflammatory cytokines. Incorporating Western blot analysis of NF-κB subunits, especially phosphorylated p65, into future studies would yield additional mechanistic insights.

## 5. Conclusions

In summary, MTX administration in rats induced marked kidney injury characterized by impaired renal function, severe oxidative and nitrosative stress, robust inflammatory activation, and mitochondrial-dependent apoptosis, all of which translated into significant structural damage to renal tissue. Piceatannol exerted a protective influence against these alterations, but its encapsulation into liposomal nanoparticles substantially amplified its efficacy. PIC-LNPs more effectively re-established antioxidant defenses, suppressed TLR4/NF-κB and MAPK signaling, reduced pro-inflammatory cytokines and nitrosative burden, and normalized key apoptotic markers, resulting in improved renal function tests and preservation of glomerular and tubular architecture. These findings point to liposomal piceatannol as a promising nanotherapeutic approach for attenuating chemotherapy-associated nephrotoxicity and support further investigation of PIC-LNPs as a potential adjunct strategy to safeguard renal function during MTX-based regimens.

## Figures and Tables

**Table 1:** Primer sequences used for RT-qPCR analysis of kidney tissue.

Gene	Sense (5′–3′)	Antisense (5′–3′)
*Nrf2*	F: TTTGTAGATGACCATGAGTCG	R: TCCTGCCAAACTTGCTCCAT
*HO-1*	F: ATGTCCCAGGATTTGTCCGA	R: ATGGTACAAGGAGGCCATCA
*NFκB*	F: AGTCCCGCCCCTTCTAAAAC	R: CAATGGCCTCTGTGTAGCCC
*TLR4*	F: ATCATCCAGGAAGGCTTCCA	R: GCTGCCTCAGCAAGGACTTC
*c-Fos*	F: CCCGTAGACCTAGGGAGGAC	R: CAATACACTCCATGCGGTTG
*c-Jun*	F: CCAACCAACGTGAGTGCAAG	R: CGTCCCCGCTTCAGTAACAA
*β-Actin*	F: CAGCCTTCCTTCTTGGGTATG	R: AGCTCAGTAACAGTCCGCCT

Nrf2: Nuclear factor erythroid 2-related factor 2, HO-1: Heme oxygenase-1, NF-κB: Nuclear factor kappa-light-chain-enhancer of activated B cells, TLR4: Toll-like receptor 4, c-FOS, cellular FBJ osteosarcoma oncogene; c-JUN: cellular JUN oncogene, β-Actin: Beta actin.

**Table 2:** Semi-quantitative histopathological scoring of renal tissue alterations.

Score	Tubular Damage and Necrosis in Kidney Sections	Structural Alterations of Glomeruli	Inflammation	Hemorrhage
0	None	None	None	None
1	Focal degenerative changes were detected in 1–2 of the 12 kidney fields analyzed.	Focal mild atrophy of glomeruli was detected in 1–2 of the evaluated kidney sections.	Only 1–2 out of 12 renal fields showed slight inflammatory cell infiltration.	Focal mild congestion and occasional hemorrhagic spots were detected in 1–2 of the analyzed kidney sections.
2	Multiple kidney fields (3–6 of 12) displayed degenerative changes characterized by focal epithelial sloughing.	Mild to moderate glomerular shrinkage was observed in 3–4 of the 12 examined kidney fields.	Focal moderate inflammatory infiltration was detected in 3–4 of the analyzed kidney sections.	Focal hemorrhage with mild interstitial congestion was detected in 3–4 of the analyzed kidney sections.
3	Severe tubular necrosis was evident in 7–9 out of 12 kidney fields analyzed.	Lamellar fusion was observed in 5–6 of the 12 kidney fields examined.	Significant inflammation was evident in 5–7 of the 12 kidney fields examined.	In 5–6 out of 12 renal fields, the interstitium exhibited pronounced congestion with focal hemorrhage.

## Data Availability

The original contributions presented in this study are included in the article/Supplementary Materials. Further inquiries can be directed to the corresponding author.

## Supplementary Materials

The following supporting information can be downloaded at: https://www.mdpi.com/article/10.3390/biom16040517/s1, Table S1. Commercial assay kits and reagents used in the study.

## Abbreviations

The following abbreviations are used in this manuscript:

8-OHdG	8-Hydroxy-2′-deoxyguanosine
ANOVA	Analysis of variance
AASLD	American Association for the Study of Liver Diseases
Bax	Bcl-2-associated X protein
Bcl-2	B-cell lymphoma-2
BM	Basement membrane
CAT	Catalase
cDNA	Complementary DNA
DAB	3,3′-Diaminobenzidine
DCFH-DA	2′,7′-Dichlorodihydrofluorescein diacetate
DCT	Distal convoluted tubule
DMSO	Dimethyl sulfoxide
DTNB	5,5′-Dithiobis(2-nitrobenzoic acid)
ELISA	Enzyme-linked immunosorbent assay
ERK	Extracellular signal-regulated kinase
FTIR	Fourier-transform infrared spectroscopy.
GPx	Glutathione peroxidase
GSH	Reduced glutathione
H&E	Hematoxylin and eosin
HO-1	Heme oxygenase-1
HRP	Horseradish peroxidase
i.p.	Intraperitoneal
IL-1β	Interleukin-1 beta
IL-6	Interleukin-6
iNOS	Inducible nitric oxide synthase
JNK	c-Jun N-terminal kinase
L-PC	L-α-phosphatidylcholine
MAPK	Mitogen-activated protein kinase
MDA	Malondialdehyde
MERC	Medical Experimental Research Center
mRNA	Messenger RNA
MTX	Methotrexate
MyD88	Myeloid differentiation primary response protein 88
NF-κB	Nuclear factor kappa-light-chain-enhancer of activated B cells
NO	Nitric oxide
Nrf2	Nuclear factor erythroid 2-related factor 2
PC	Protein carbonyl
PCT	Proximal convoluted tubule
PBS	Phosphate-buffered saline
PIC	Piceatannol
PIC-LNPs	Piceatannol-loaded liposomal nanoparticles
PDI	Polydispersity index
RFU	Relative fluorescence units
ROS	Reactive oxygen species
SOD	Superoxide dismutase
TBARS	Thiobarbituric acid reactive substances
TEM	Transmission electron microscopy
TLR4	Toll-like receptor 4
TNF-α	Tumor necrosis factor-alpha
