# Supplementary material for: Liposomal Piceatannol Mitigates Methotrexate-Induced Oxidative Renal Injury via Modulation of Nrf2/HO-1, TLR4/NF-κB, MAPK, and Apoptotic Pathways in Rats
Source: Biomolecules. 2026 Mar 31;16(4):517. doi: 10.3390/biom16040517 (PMC13114049; doi:10.3390/biom16040517)
Supplement: Supplementary file 1 [file biomolecules-16-00517-s001.zip › biomolecules-4229854-supplementary.pdf]

Liposomal Piceatannol Mitigates Methotrexate-Induced Oxidative Renal Injury via Modulation of Nrf2/HO-1, TLR4/NF-κB, MAPK, and Apoptotic Pathways in Rats

Table S1. Commercial assay kits and reagents used in the study

Analyte/Use	Assay type/ principle	Supplier	Catalogue No.
GSH	Colorimetric, DTNB reaction	BioDiagnostic, Cairo, Egypt	2511
SOD	Colorimetric, inhibition of NBT reduction	BioDiagnostic, Cairo, Egypt	2521
GPx	Colorimetric, NADPH-coupled assay	BioDiagnostic, Cairo, Egypt	2524
CAT	Colorimetric, H_2O_2 decomposition	BioDiagnostic, Cairo, Egypt	2517
MDA	TBARS assay	BioDiagnostic, Cairo, Egypt	MD 2529
Intracellular ROS	DCFH-DA-based fluorescence	MyBioSource, San Diego, CA, USA	MBS2540517
Protein carbonyl (PC)	Sandwich ELISA	MyBioSource, San Diego, CA, USA	MBS760520
8-OHdG	Sandwich ELISA	MyBioSource, San Diego, CA, USA	MBS732375
Nrf2	Sandwich ELISA	MyBioSource, San Diego, CA, USA	MBS3807961
HO-1	Sandwich ELISA	MyBioSource, San Diego, CA, USA	MBS2024438
TNF-α	Sandwich ELISA	MyBioSource, San Diego, CA, USA	MBS282960
IL-1β	Sandwich ELISA	MyBioSource, San Diego, CA, USA	MBS232385
TLR4	Sandwich ELISA	MyBioSource, San Diego, CA, USA	MBS161614
Total NO metabolites	Colorimetric microplate assay	MyBioSource, San Diego, CA, USA	MBS8243214
Bax	Sandwich ELISA	MyBioSource, San Diego, CA, USA	MBS2512405
Bcl-2	Sandwich ELISA	MyBioSource, San Diego, CA, USA	MBS457882
Caspase-3	Sandwich ELISA	MyBioSource, San Diego, CA, USA	MBS743552
Nuclear NF-κB p65	Transcription factor ELISA (oligonucleotide-capture based)	Abcam, Cambridge, UK	ab133112
Nuclear protein extraction	Nuclear extraction kit	Abcam, Cambridge, UK	ab113474
p-ERK1/2	SimpleStep ELISA	Abcam, Cambridge, UK	ab176660
p-p38 MAPK	SimpleStep ELISA	Abcam, Cambridge, UK	ab176664
p-JNK	SimpleStep ELISA	Abcam, Cambridge, UK	ab176662
